# Stable predictive markers for *Phytophthora sojae* avirulence genes that impair infection of soybean uncovered by whole genome sequencing of 31 isolates

**DOI:** 10.1186/s12915-018-0549-9

**Published:** 2018-07-26

**Authors:** Geneviève Arsenault-Labrecque, Humira Sonah, Amandine Lebreton, Caroline Labbé, Geneviève Marchand, Allen Xue, François Belzile, Brian J. Knaus, Niklaus J. Grünwald, Richard R. Bélanger

**Affiliations:** 1 Département de Phytologie, Université Laval, Québec, QC Canada; 2 Agriculture and Agri-Food Canada, Harrow, ON Canada; 3 Agriculture and Agri-Food Canada, Ontario, ON Canada; 4 Horticultural Crops Research Laboratory, USDA Agricultural Research Service, Corvallis, OR USA

**Keywords:** Avr genes, Effectors, Genomics, Haplotype analysis, Oomycete, *Phytophthora sojae*, Plant pathogen, R genes

## Abstract

**Background:**

The interaction between oomycete plant pathogen *Phytophthora sojae* and soybean is characterized by the presence of avirulence (*Avr*) genes in *P. sojae*, which encode for effectors that trigger immune responses and resistance in soybean via corresponding resistance genes (*Rps*). A recent survey highlighted a rapid diversification of *P. sojae Avr* genes in soybean fields and the need to deploy new *Rps* genes. However, the full genetic diversity of *P. sojae* isolates remains complex and dynamic and is mostly characterized on the basis of phenotypic associations with differential soybean lines.

**Results:**

We sequenced the genomes of 31 isolates of *P. sojae*, representing a large spectrum of the pathotypes found in soybean fields, and compared all the genetic variations associated with seven *Avr* genes (1a, 1b, 1c, 1d, 1k, 3a, 6) and how the derived haplotypes matched reported phenotypes in 217 interactions. We discovered new variants, copy number variations and some discrepancies with the virulence of previously described isolates with *Avr* genes, notably with *Avr1b* and *Avr1c*. In addition, genomic signatures revealed 11.5% potentially erroneous phenotypes. When these interactions were re-phenotyped, and the *Avr* genes re-sequenced over time and analyzed for expression, our results showed that genomic signatures alone accurately predicted 99.5% of the interactions.

**Conclusions:**

This comprehensive genomic analysis of seven *Avr* genes of *P. sojae* in a population of 31 isolates highlights that genomic signatures can be used as accurate predictors of phenotypes for compatibility with *Rps* genes in soybean. Our findings also show that spontaneous mutations, often speculated as a source of aberrant phenotypes, did not occur within the confines of our experiments and further suggest that epigenesis or gene silencing do not account alone for previous discordance between genotypes and phenotypes. Furthermore, on the basis of newly identified virulence patterns within *Avr1c*, our results offer an explanation why *Rps1c* has failed more rapidly in the field than the reported information on virulence pathotypes*.*

**Electronic supplementary material:**

The online version of this article (10.1186/s12915-018-0549-9) contains supplementary material, which is available to authorized users.

## Background

*Phytophthora sojae* (Kauf. & Gerd.), a hemibiotrophic oomycete causing root and stem rot in soybean, is among the top 10 plant-pathogenic oomycetes/fungi of both scientific and economic importance [1]. Management of *P. sojae* relies mostly on the development of cultivars with major resistance (*Rps*) genes. The development of root and stem rot caused by *P. sojae* is determined by the gene-for-gene relationship between resistance (*Rps*) genes in soybean and their matching avirulence (*Avr*) genes in the pathogen. Typically, *Rps* genes encode, or are predicted to encode for proteins having nucleotide-binding site and leucine-rich repeat (NLR receptors) while *P. sojae Avr* genes code for small effector proteins mostly with RXLR and DEER amino acid motifs. In such cases, NLR receptors from soybean recognize the RXLR effectors encoded by *Avr* genes from *P. sojae*, inducing an appropriate defense response [2, 3]. The pathogen can avoid recognition conferred by *Rps* genes through various mutations such as a substitutions, frameshift mutations, partial or complete deletions, large insertions, recombinations, or changes in expression of *Avr* genes [4].

To date, over 27 major *Rps* genes have been identified in soybean [2] and about 12 *Avr* genes have been identified and characterized in *P. sojae* [5–9]*.* Most of the *Avr* genes are clustered together on *P. sojae* chromosomes, and many of them are candidate paralogs. For instance, *Avr1a* and *Avr1c* have very similar sequences [10]. In addition, some of the gene pairs earlier thought to be different genes, such as *Avr3a*/*Avr5* and *Avr6*/*Avr4*, turned out to be different alleles of the same gene [11, 12]. In the case of *Avr1a*, deletion of two out of four nearly identical copies of the gene has been found to cause virulence. Similarly, some *P. sojae* strains have as many as four paralogs of *Avr3a*, and some have only one [13]. Such high levels of similarity, tandem duplications, and variation in the number of copies make it very difficult to develop sequence-based diagnostic markers.

Avirulence (*Avr*) genes from *Phytophthora* species are mostly located in highly dynamic genome areas containing duplications and repetitive sequences that are prone to chromosomal rearrangements [4]. Characterization of such loci needs high-quality sequencing with “border” coverage and high depth. High levels of sequence variation, duplications, interdependency of *Avr* genes, and rapid evolution complicate the task of characterizing newly evolved strains. With approximately 20.5 million metric ton losses attributed to Phytophthora root and stem rot since 1996, efficient tools to rapidly and accurately identify virulence features in *P. sojae* have become essential to prevent disease outbreaks [14]. In this regard, recent advances in sequencing technology provide the opportunity to perform whole genome sequencing (WGS) of multiple strains. This approach facilitates the identification of all potential variations, and chromosomal rearrangements, and can be used for the identification of variation signatures (haplotypes) associated with virulence factors [15]. Haplotypes representing the allelic variation of a given gene have also been found to be tightly linked with the copy number variation and expression of the same gene [15–17]. Na et al. [10] identified *Avr1a* and *Avr1c* as a pair of tandem duplicated genes near the *Avr1c* locus by using a WGS approach.

Apart from the need for high-quality sequencing to decipher *Avr* genes, precise phenotyping of the interactions between pathotypes and differentials remains an essential component to assess the functionality of either *Avr* or *Rps* genes. For this purpose, several phenotyping methods have been developed and proposed [18–23]. Over the years, the hypocotyl inoculation test has become the standard test, particularly because of its ease of use [24]. However, as convenient as the hypocotyl inoculation method is, it has limitations leading to the identification of false positives or negatives [25], which can bring confusion about the presence and/or functionality of *Avr* genes in *P. sojae* isolates. Recently, Lebreton et al. [26] used a simplified hydroponic assay to more robustly characterize the phenotypes by inoculating the root system of soybean plants directly with zoospores of *P. sojae*. It thus offers a potentially better option to link phenotypes with genotypes of tested *P. sojae* isolates.

In the present study, a diverse set of 31 *P. sojae* isolates representing the range of pathotypes commonly observed in soybean fields was sequenced using WGS. To understand the evolution and genetic constitution of *P. sojae* strains, haplotype analyses using the WGS data were performed for the seven most important *Avr* genes found in *P. sojae* populations: 1a, 1b, 1c, 1d, 1k, 3a, and 6. Our data provide new insights into the complexity of *Avr* genes and their associated functionality and reveal that their genomic signatures can be used as accurate predictors of phenotypes for interaction with *Rps* genes in soybean.

## Results

### Sequencing and mapping

A total of 852,950,094 reads were obtained from paired-end sequencing of the 31 *P. sojae* isolates on the Illumina HiSeq 2500 sequencer. The number of sorted raw sequence reads per isolate ranged from 15 to 52 M reads with an average of 27 M reads per isolate, with a mean Phred-score of 32.4. Reads were processed using Trimmomatic, and the processed reads were mapped to the reference genome [27]. For every isolate, more than 96% of the reads were accurately mapped to the reference genome with a mean depth coverage of 68×.

### Coverage, distribution, and predicted functional impact of SNPs

The HaplotypeCaller pipeline from GATK retained 260,871 variants among the 31 isolates. Stringent filtering of the variants based on sequence depth and mapping quality using vcfR retained a total of 204,944 high-quality variants. Variant analysis with SnpEff tool [28] identified 172,143 single nucleotide polymorphisms (SNPs), 14,627 insertions, and 18,174 small indels in the total number of variants. Variants in coding regions were categorized as synonymous and non-synonymous substitutions; 61.1% of the SNPs resulted in a codon that codes for a different amino acid (missense mutation; 59.5%) or the introduction of a stop codon (nonsense mutation 1.6%), whereas the remaining 38.9% of the SNPs were considered to be synonymous mutations.

### Phylogenetic analysis

**Fig. 1:**
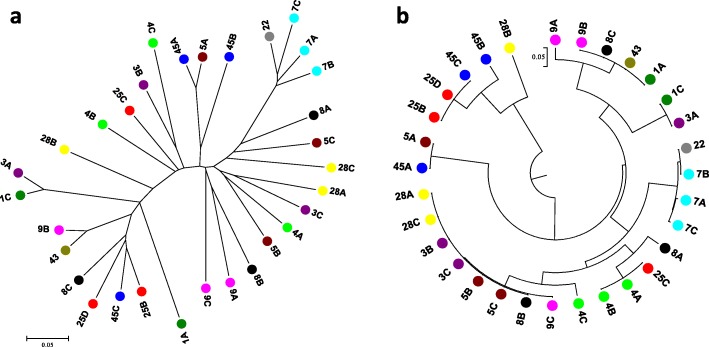
Phylogenetic analysis from variants among 31 isolates of *Phytophtora sojae*. **a** Neighbor-joining tree using whole-genome data. **b** Neighbor-joining tree using variants within seven *Avr* genes region (1a, 1b, 1c, 1d, 1k, 3a, 6). Each isolate is color-coded based on its initial virulence profile (from the hypocotyl test: see Table 2)

A phylogenetic tree was first constructed with all 204,944 variants among the 31 isolates. Results showed that, based on whole-genome data, no general inference could be made on the relationship between the virulence profiles of all isolates and their genetic variability, except for those of race 7 (Fig. 1a). A second phylogenetic tree was then constructed with variants belonging only to the seven *Avr* genes used to define those virulence profiles (Fig. 1b). This highlighted a certain level of clustering based on the virulence profile of the isolates while some discrepancies were noted. For example, isolates 25C did not cluster with other isolates from the same virulence profile (25B and 25D) or isolates from race 8 (8A, 8B, and 8C) were all found on different branches of the tree. Links among the seven *Avr* genes were then further investigated on the basis of haplotype analysis.

### Haplotypes for *Avr1a*

**Fig. 2:**
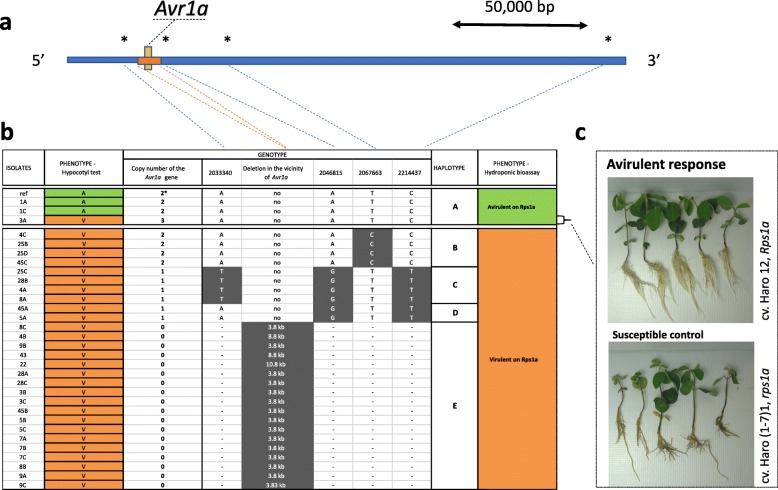
Structural and nucleotide diversity at the *Avr1a* locus among 31 isolates of *Phytophthora sojae* reveal distinct haplotypes associated with virulence phenotypes. **a** Variants in the vicinity of the *Phytophthora sojae Avr1a* gene. The yellow box represents the coding region of the gene. The orange box shows the location of the deletion. Asterisks (*) indicate approximate positions of the SNPs. Those SNPs are representative of a cluster of SNPs defining a haplotype. **b** Schematic graph of the position of the SNPs for each isolate, grouped by haplotypes. SNPs in gray background are different from the reference genome (isolate P6497). **c** Phenotypic response of the outliers (when the phenotype did not match the genotype based on the hypocotyl test) from the hydroponic assay. Responses showed here are representative of all isolates tested. All phenotypic responses of other tested isolates can be found in Additional file 2. CNV of *Avr1a* gene for the reference genome (P6497) is based on results from Qutob et al. [13]

For all 31 isolates, CNV was analyzed based on depth of coverage and, for *Avr1a*, it ranged between zero and three copies (Fig. 2b). Among isolates with zero copy, all were virulent on *Rps1a*. For the remaining isolates, no SNPs or indels were observed within the coding region of *Avr1a* (Fig. 2a). However, we observed SNPs flanking *Avr1a* that were in high linkage disequilibrium (LD) (*R*^2^ ≥ 0.7) and defined four distinct haplotypes (Fig. 2b). Additional variants were also found but did not offer a higher level of discrimination (Additional file 1). All isolates sharing three of these (B, C, and D) were virulent on *Rps1a* while among isolates with haplotype A, all but isolate 3A were incompatible based on the hypocotyl assay. After re-phenotyping this isolate with the hydroponic bioassay, it was characterized as being unable to infect the differential carrying *Rps1a* confirming that haplotype A was the only one associated with an incompatible interaction with *Rps1a* (Fig. 2c).

### Haplotypes for *Avr1b*

**Fig. 3:**
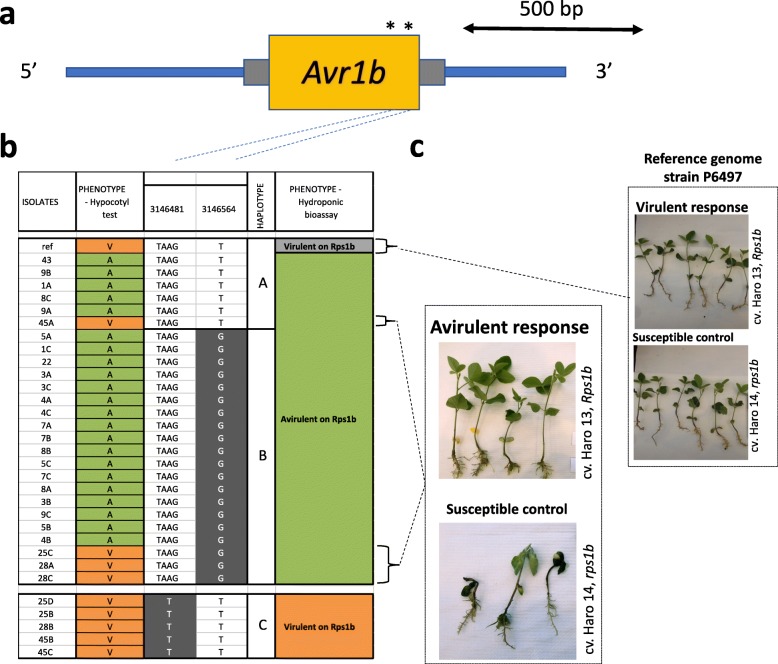
Nucleotide diversity at the *Avr1b* locus among 31 isolates of *Phytophthora sojae* reveal distinct haplotypes associated with virulence phenotypes. **a** Variants within the coding region of the *Phytophthora sojae Avr1b* gene. Yellow box represents the coding region of the gene and gray bars, 5′ and 3′ UTR. Asterisks (*) indicate approximate positions of the SNPs and small indels. Those variants are representative of a cluster of variants defining a haplotype. **b** Schematic graph of the position of the SNPs for each isolate, grouped by haplotypes. Variants in gray background are different from the reference genome (isolate P6497). **c** Phenotypic response of the reference genome strain (P6497) and the outliers (when the phenotype did not match the genotype based on the hypocotyl test) from the hydroponic assay. Responses showed here are representative of all isolates tested. All phenotypic responses of other tested isolates can be found in Additional file 3

No CNVs or deletions were observed for *Avr1b* (Fig. 3a). Within the coding region of the gene, 17 variants were observed: 14 missense variants (SNPs), two small indels of three nucleotides each, and one synonymous SNP (Additional file 1). None of these variants were predicted to have a high functional impact. Based on the LD between these variants, two tag variants were retained and defined three haplotypes (Fig. 3b). Most isolates of haplotypes A and B were avirulent while all isolates with haplotype C were virulent. Among haplotypes A and B, four isolates with a discordant phenotype were re-tested with the hydroponic assay and were found to be avirulent to *Rps1b* (Fig. 3c), confirming haplotypes A and B as being associated with an incompatible interaction with *Rps1b* (Fig. 3b) in our study. To verify that the genotype of these four isolates had not changed over time, we re-sequenced the *Avr1b* region of these isolates together with representative isolates from each haplotype group and P6497 and confirmed the same mutations. On the other hand, P6497 used for the reference genome, associated with haplotype A, did show a phenotype of virulence when assayed in the hydroponic system (Fig. 3c).

### Haplotypes for *Avr1c*

**Fig. 4:**
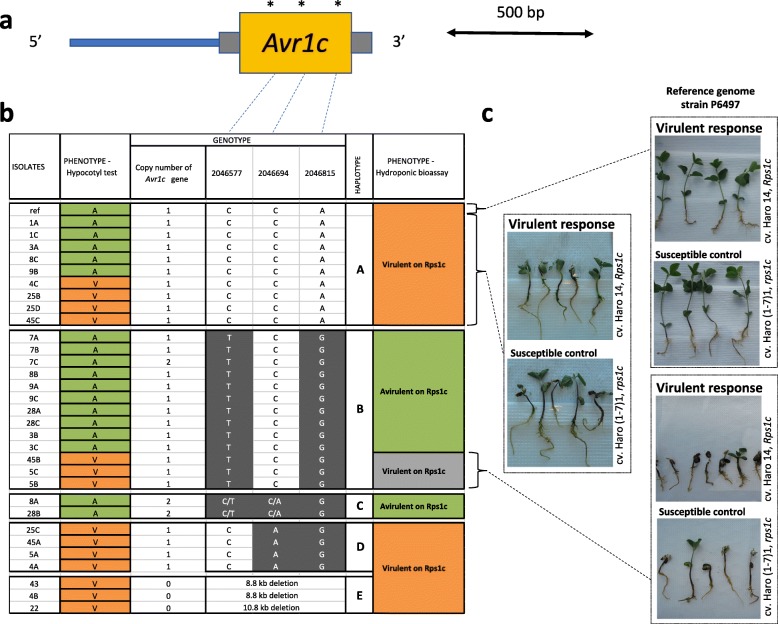
Structural and nucleotide diversity at the *Avr1c* locus among 31 isolates of *Phytophthora sojae* reveal distinct haplotypes associated with virulence phenotypes. **a** Variants within the coding region of the *Phytophthora sojae Avr1c* gene. Yellow box represents the coding region of the gene and gray bars, 5′ and 3′ UTR. Asterisks (*) indicate approximate positions of the SNPs. Those SNPs are representative of a cluster of SNPs defining a haplotype. **b** Schematic graph of the position of the SNPs for each isolate, grouped by haplotypes. SNPs in gray background are different from the reference genome (isolate P6497). **c** Phenotypic response of reference genome strain (P6497) and the outliers (when the phenotype did not match the genotype based on the hypocotyl test) from the hydroponic assay. Responses showed here are representative of all isolates tested. All phenotypic responses of other tested isolates can be found in Additional file 3

Copy number variation was observed for *Avr1c*; complete deletion of the *Avr1c* gene was observed in three isolates while others presented one or two copies of the gene (Fig. 4b). Interestingly, this deletion is the same reported earlier for the *Avr1a* gene that immediately flanks *Avr1c* (Figs. 2b and 4b). The remaining isolates presented a total of 24 variants within the coding region of the gene; two were synonymous while the rest were missense mutations, none of which being predicted to have a high functional impact (Additional file 1). After removal of redundant markers (based on LD), a total of four tag variants defined four haplotypes (A to D; Fig. 4b). Haplotypes C and D were shared by isolates that had a consistent phenotype, avirulent and virulent, respectively (Fig. 4b). Haplotype C was also the only haplotype to present a majority of heterozygous SNPs. In contrast, haplotype A was shared by five isolates previously phenotyped as avirulent to *Rps1c* and four phenotyped as being virulent. All nine isolates were re-phenotyped in the hydroponic assay, and the results showed a clear association with virulence to *Rps1c* (Fig. 4c). In addition, P6497 (ref) associated with haplotype A and a phenotype of avirulence was found to be virulent to *Rps1c* when phenotyped in the hydroponic assay. For haplotype B, most isolates were phenotyped as avirulent to *Rps1c*, with the exception of three isolates (5B, 5C, and 45B) originally labeled as virulent. Variants within a 1-kb upstream or downstream region of the gene could not define new haplotypes for these three outliers. These three isolates were re-phenotyped using the hydroponic bioassay and were still characterized as virulent (Fig. 4c). To further investigate the cause of this discrepancy, the *Avr1c* region for representative isolates from each haplotype group, including initial outliers from haplotype A, was re-sequenced using Sanger sequencing and confirmed the same mutations.

**Fig. 5:**
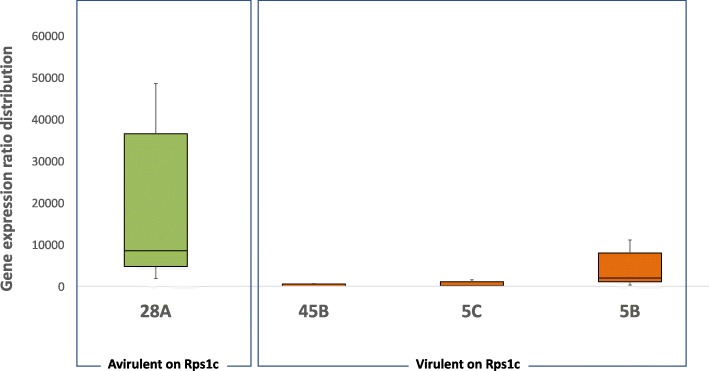
Relative expression of the *Phytophthora sojae Avr1c* gene measured by real-time quantitative PCR in avirulent and virulent isolates. Fold change was based on gene expression ratio with virulent isolate 4C. Actin was used as an internal control to normalize gene expression. Bars represent standard error from the mean (*n* = 4). Individual values for every samples can be found in Additional file 5

In order to determine if differences in gene expression could explain the aberrant phenotype, a qPCR-based gene expression analysis was performed. Interestingly, the expression of *Avr1c* in isolates 5B, 5C, and 45B was significantly lower than that in the avirulent isolate, 28A, which would explain their virulence (Fig. 5). Attempts were then made to find distant variants associated with lower expression via genome-wide sequence comparison. A total of 690 unique mutations, present in isolate 5B and absent in all other isolates of the same haplotype, were identified. Most of these were in non-coding regions, but five frameshift variants and two inframe deletions were observed, including a deletion of 29 bases in the *Avh220* gene (coding for an effector) were found to be unique to 5B. For isolate 5C, a total of 473 unique mutations were observed including a 9-bp deletion in the Sin3 transcription factor that was unique to this isolate (Additional file 2). Finally, for isolate 45B, over 1000 unique mutations were observed including four in-frame deletions and ten stop/gain mutations. However, none could be clearly linked with the lower expression of *Avr1c* in this isolate.

### Haplotypes for *Avr1d*

**Fig. 6:**
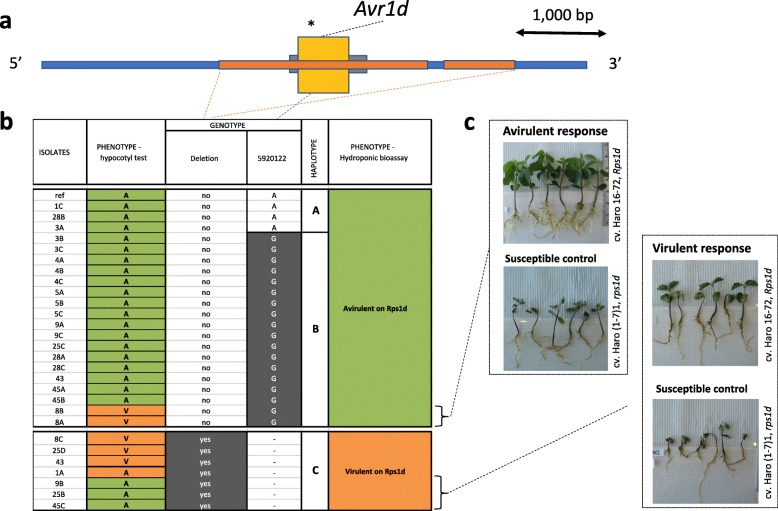
Structural and nucleotide diversity at the *Avr1d* locus among 31 isolates of *Phytophthora sojae* reveal distinct haplotypes associated with virulence phenotypes. **a** Deletion in the vicinity of the *Phytophthora sojae Avr1d* locus. Yellow box represents exon and gray bars, 5′ and 3’ UTR. Orange boxes show the position of deletions in virulent isolates. **b** Schematic graph of the genotypes based on the deletion. Genotypes in gray background are different from the reference genome (isolate P6497). **c** Phenotypic response of the outliers (when the phenotype did not match the genotype based on the hypocotyl test) from the hydroponic assay. Responses showed here are representative of all isolates tested. All phenotypic responses of other tested isolates can be found in Additional file 3

A complete deletion of the *Avr1d* gene was observed for seven isolates (Fig. 6b). The deletion encompassed both the upstream and downstream regions of the gene for a total deletion size of 2.3 kb, with another upstream deletion of 0.8 kb, separated by a segment of 177 bp (Fig. 6a). The remaining isolates presented one copy of the gene, and 21 variants were observed within the coding region: one was synonymous while the others were missense variants, none of which were predicted to have a high functional impact (Additional file 1). Based on LD, one tag variant was retained and two haplotypes (A and B) could be defined. Genomic data coincided with the original phenotypes based on the hypocotyl assay in 25 out of 31 interactions. However, from the original phenotyping by Xue et al. [29], two isolates predicted to be avirulent based on the genotype were phenotyped as virulent and four isolates predicted as virulent were phenotyped as avirulent. When these isolates were phenotyped with the hydroponic assay, all the isolates with a predicted genotype of virulence were consistenly associated with virulence while the isolate expected to be avirulent based on the haplotype was phenotypically avirulent, confirming that deletion of *Avr1d* is consistently linked to virulence (Fig. 6).

### Haplotypes for *Avr1k*

**Fig. 7:**
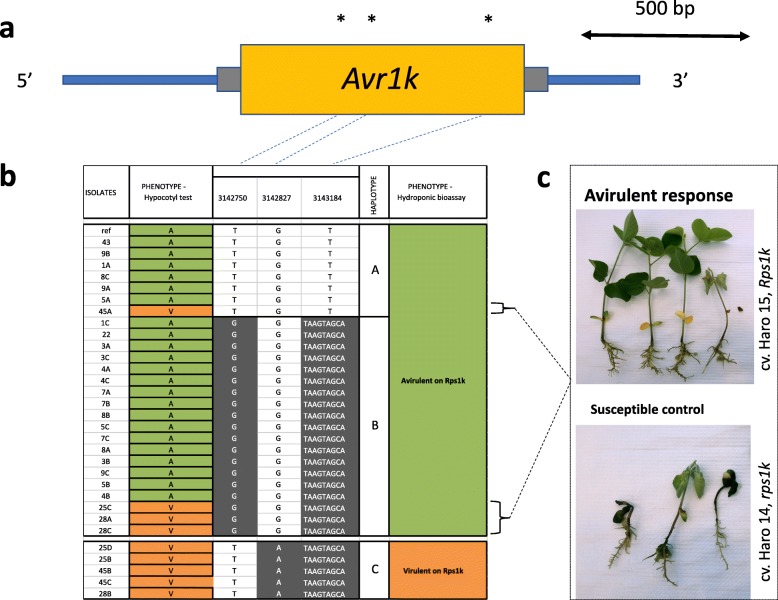
Nucleotide diversity at the *Avr1k* locus among 31 isolates of *Phytophthora sojae* reveal distinct haplotypes associated with virulence phenotypes. **a** Variants within the coding region of the *Phytophthora sojae Avr1k* gene. Yellow box represents the coding region of the gene and gray bars, 5′ and 3′ UTR. Asterisks (*) indicate approximate positions of the SNPs and small indel. Those variants are representative of a cluster of variants defining a haplotype. **b** Schematic graph of the position of the variants for each isolate, regrouped by haplotypes. Variants in gray background are different from the reference genome (isolate P6497). **c** Phenotypic response of the outliers (when the phenotype did not match the genotype based on the hypocotyl test) from the hydroponic assay. Responses showed here are representative of all isolates tested. All phenotypic responses of other tested isolates can be found in Additional file 3

No CNVs or deletions were observed for *Avr1k* (Fig. 7a). Inside the genic region, 16 variants were found: one synonymous variant, 14 missense variants, and one deletion of eight nucleotides causing a frameshift in the ORF and leading to a premature stop codon towards the 3′ end of the gene (Additional file 1). This latter variant is the only one considered to have a high impact on the functionality of the gene. The three tag variants within the gene (based on LD) formed three distinct haplotypes (Fig. 7b). As observed previously for *Avr1b*, the first two haplotypes (A and B) contained all the isolates avirulent to *Rps1k* plus four isolates previously phenotyped as virulent to *Rps1k* with the hypocotyl test. Interestingly, the exact same outliers gave an initial phenotype of virulence with *Avr1b*. To verify that the genotype of these outliers had not changed over time, the *Avr1k* gene region was re-sequenced for these isolates and showed the same mutations as observed by WGS. Haplotype C only contained isolates virulent to *Rps1k*. Re-phenotyping of the four outliers confirmed their incompatibility with *Rps1k* as shown in Fig. 7c. The eight-nucleotide frameshift mutation leading to an early stop codon was found in both haplotypes B and C, although the former was associated with an avirulent phenotype and the latter with a virulent one.

### Haplotypes for *Avr3a*

**Fig. 8:**
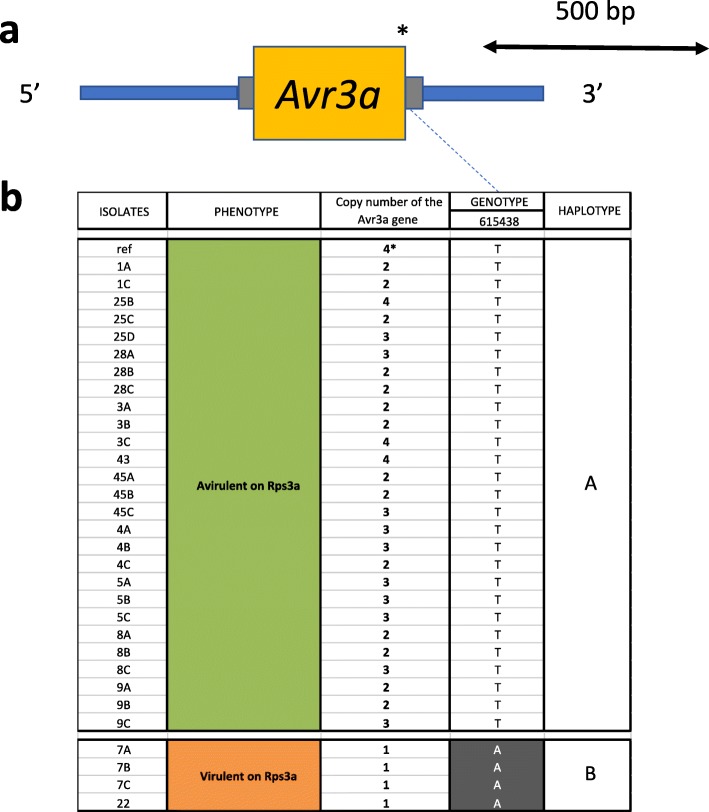
Structural and nucleotide diversity at the *Avr3a* locus among 31 isolates of *Phytophthora sojae* reveal distinct haplotypes associated with virulence phenotypes. **a** Variants in the coding region of the *Phytophthora sojae Avr3a* region. Yellow box represents the coding region of the gene and gray bars, 5′ and 3′ UTR. Asterisk (*) indicate approximate positions of the SNPs and small indel. Those variants are representative of a cluster of variants defining a haplotype. **b** Schematic graph of the position of the variants for each isolate, regrouped by haplotypes. Variants in gray background are different from the reference genome (isolate P6497). Phenotype results were confirmed by re-testing a number of isolates with the hydroponic assay (Additional file 3). CNV of *Avr3a* gene for the reference genome (P6497) is based on data from Qutob et al. [13]

Copy number variation was observed between isolates, ranging from one to four copies; all isolates virulent to *Rps3a* contained one copy of the gene, while all avirulent isolates had two to four copies (Fig. 8b). Furthermore, we observed 15 variants in the coding region of the *Avr3a* gene, including one inframe deletion of six nucleotides and 14 SNPs, of which two were synonymous variants, 11 were missense variants, and one caused the loss of the stop codon (Additional file 1). Only the latter variant is considered to have a high impact on the functionality of the gene. All those variants were homozygous suggesting that for isolates with multiple copies of the Avr3a gene, every copy shares the same allele. Based on the retained tag variant, two distinct haplotypes were observed. Haplotype A was consistently associated with an incompatible interaction with *Rps3a* while haplotype B was associated with a compatible one (Fig. 8b).

### Haplotypes for *Avr6*

**Fig. 9:**
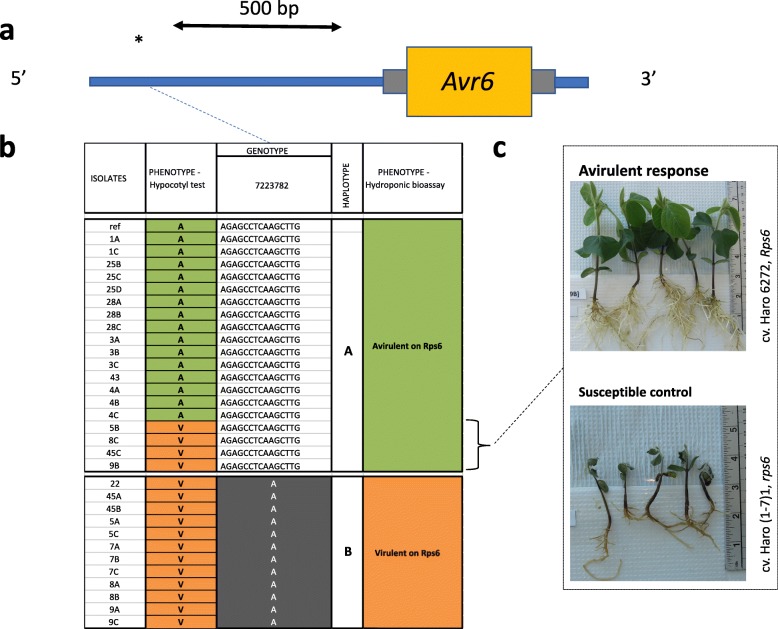
Structural and nucleotide diversity at the *Avr6* locus among 31 isolates of *Phytophthora sojae* reveal distinct haplotypes associated with virulence phenotypes. **a** Variants in the upstream region of the *Phytophthora sojae Avr6* gene. Yellow box represents exon and gray bars, 5′ and 3′ UTR. Asterisks (*) indicate approximate positions of the SNPs and small indel. **b** Schematic graph of the position of the variants for each isolates, regrouped by haplotypes. Variants in gray background are different from the reference genome (isolate P6497). **c** Phenotypic response of the outliers (when the phenotype did not match the genotype based on the hypocotyl test) from the hydroponic assay. Responses showed here are representative of all isolates tested. All phenotypic responses of other tested isolates can be found in Additional file 3

No CNVs or deletions were observed for the *Avr6* gene (Fig. 9a). Furthermore, no variants were found within the coding region of *Avr6*, but five were found in the upstream region of the gene. From these, four were SNPs, and one was a deletion of 15 nucleotides, but none of them were predicted to have a high functional impact (Additional file 1). A visual inspection of these variants revealed two distinct haplotypes, represented by one tag variant in Fig. 9b. All isolates incompatible with *Rps6* based on the hypocotyl test were associated with haplotype A, as well as four isolates initially phenotyped as virulent. These four isolates were found to be avirulent to *Rps6* via the hydroponic assay (Fig. 9c). Isolates corresponding to haplotype B were consistently associated with a compatible interaction.

## Discussion

This work describes an analysis of the genetic variations of seven *P. sojae* avirulence genes through whole genome sequencing of 31 isolates in an effort to understand and explain their interaction with *Rps* genes. Through improved re-phenotyping, evaluation of sequence stability over time, expression analysis, and genome-wide sequence comparisons, we define new variants, copy number variations, and potential new factors of virulence of *P. sojae*. We further provide evidence that one haplotype of *Avr1c* from the reference genome is likely associated to a different phenotype. Taken together, our results showed that genomic signatures alone accurately predicted 216 of the 217 (99.5%) phenotype interactions studied, and that those signatures remained stable over time.

In the specific context of the *P. sojae*-soybean interaction, very little attention has been placed on the accuracy and reproducibility of phenotypic procedures when studying the interaction of avirulence and resistance genes. This situation may lead to erroneous inferences with regard to the nature of avirulence genes or the mechanisms explaining gain of virulence, as highlighted in this study. With 31 isolates interacting with seven different *Rps* genes from soybean, we had a total of 217 interactions to consider that linked the haplotype with the original phenotyping result from the hypocotyl test. The hypocotyl inoculation method has long been used for characterizing pathotypes of *P. sojae* isolates but has also encountered some limitations in the past where re-testing gave variable results in terms of virulence profiles, leading to a rate of 10–20% of false positives or negatives [25]. In our study, 26 out of 217 interactions were initially inconsistent with the observed genotype. We re-phenotyped these using a recently described hydroponic assay [26] and found that 23 out of 26 inconsistent interactions had been incorrectly phenotyped. In addition, we highlighted an incorrect phenotype for *Avr1c* in the reference isolate P6497. Interestingly, most of the incorrect phenotypes were false positives, namely with *Avr1a*, *Avr1b*, *Avr1k*, and *Avr6*, indicating that the hypocotyl assay, bypassing the root system, is possibly too stringent. Genetic drift has also been proposed to explain virulence inconsistency of isolates over time [30], but targeted re-sequencing results of all the tested outliers, and of the concerned *avr* gene region—*Avr1c*—for the three remaining outliers (3 out of 26), showed no genetic variation compared to the whole-genome sequences, ruling out the possibility of any change by mutation or contamination within the confines of our experiments (from 2015 to 2017). Considering that of these three outliers, two are potentially explained by genomic features (distant variants putatively affecting an *Avr* gene in trans), this means that 216 out of 217 interactions were accurately predicted based on genomic signatures. In previous studies, expression polymorphism based on RT-PCR analysis was considered the next step to explain the gain of virulence mechanisms when the haplotype did not match the phenotype. However, downregulation of transcripts failed to explain all situations. For instance, Na et al. [10] and Shan et al. [31] observed the expression of an avirulence gene for a *P. sojae* isolate with a phenotype of virulence in the case of *Avr1a*, *Avr1c*, and *Avr1b*, respectively. In these cases, it was hypothesized that other effectors or epistatic effects could be responsible for these incongruent results [10]. While we cannot rule out the possibility of these genetic events, our study rather showed that an incorrect phenotype was the main source of discrepancy between the haplotype of *Avr* genes and the phenotype of *P. sojae* isolates. The use of the hydroponic test by Lebreton et al. [26] allowed rectification of these phenotyping inaccuracies and in particular elimination of false positives.

For most of the avirulence genes we studied, there were many variants representing the diversity of virulence profiles inherent to the *P. sojae* isolates. Many of the *Avr* effectors we observed had been described by other groups [3, 10–13, 31]. When we compared our data with haplotype analyses from these earlier studies, robust associations could confirm many patterns and resolve discrepancies between both the phenotypes reported previously and the new findings revealed by our analyses.

For *Avr1a*, we noticed that the complete deletion of the gene was not the sole factor that accounted for virulence of *P. sojae* to *Rps1a*. Indeed, while the absence of the gene always conferred virulence, as many as 10 isolates still displayed a phenotype of virulence without a deletion. In an earlier study, Na et al. [10] also observed the presence of *Avr1a* in virulent isolates and attributed this phenomenon to gene silencing. In this work, we were able to identify new SNPs outside the *Avr*1a gene region that discriminated between avirulent and virulent isolates. While the functional impact of these SNPs remains unknown, it will be interesting to determine if they indeed lead to silencing of *Avr1a* [10, 13] or if they affect another gene involved in the virulence to *Rps1a*. Our data have also further refined the extent of the deletion for *Avr1a*, showing that it can be as large as 10.8 kb, in which case it also encompassed *Avr1c*. Another interesting observation was the variation in the number of copies of *Avr1a* among the isolates. In a previous study, Qutob et al. [13] identified a tandem array of two identical copies of *Avr1a* and established a link between virulence and deletion of both copies, although a few isolates were virulent in spite of the presence of the gene. Within the population of 31 isolates studied, we found that the copy number could be as high as three in more than 50% of the isolates and included isolates displaying a phenotype of virulence. However, in the latter cases, we identified haplotypes associated with this phenotype of virulence to *Rps1a*.

With respect to *Avr1b*, our results identified three distinct haplotypes among the 31 isolates. More importantly, all our tested isolates with haplotype A had an incompatible interaction with differentials carrying *Rps1b* or *Rps1k*. This contrasts with data for isolate P6497, which possesses the same haplotype, but has been reported as virulent to *Rps1b* (and avirulent to *Rps1k*), based on the hypocotyl or infiltration tests [31], a phenotype confirmed in this study by the hydroponic assay. Given the possible different genetic background between our isolates and isolate P6497, we could also hypothesize that epistasic interactions leading to differences in gene expression as observed by Shan et al. [31] might be responsible for the different virulence profile of P6497. Table 1 presents a comparative analysis of the phenotypes attributed to the haplotypes found in Shan et al. [31] compared to our data. Because *Avr1b* and *Avr1k* are tightly linked [8], and *Avr1b* can also determine virulence to *Rps1k* [3], the table presents the phenotype to *Rps1b* and *Rps1k* linked to the haplotype. Haplotype I from Shan et al. [31] comprised isolates with different virulence profiles (virulent/avirulent to *Rps1b* and *Rps1k*). In our case, all isolates with haplotype A, corresponding to haplotype I, were avirulent to *Rps1b* and *Rps1k* following re-phenotyping except for isolate P6497. Incidentally, Shan et al. (2004) also observed a pattern of virulence with P6497 as well as an avirulent isolate with the same haplotype and attributed the differences to a higher expression of *Avr1b* in the latter isolate, stimulated or stabilized by another elusive gene called *Avr1b-2*. The other two haplotypes, B and C, revealed from our data correspond to haplotype II and IV from the previous study, and the phenotypes associated with them are identical. The fourth haplotype described by Shan et al. [31] and missing from our isolates, haplotype III, was associated with a rare pattern of virulence to *Rps1b* and avirulence to *Rps*1k.

**Table 1:** Comparison of haplotypes/phenotypes of 31 isolates of *Phytophthora sojae* evaluated in this study compared to data from Shan et al. [31]

This study			Shan et al., 2004		
Haplotype	Virulence		Haplotype	Virulence	
	*Rps1b*	*Rps1k*		*Rps1b*	*Rps1k*
A	A (6/6)^a^	A (6/6)	I	V (3/4)	A (3/4)
B	A (20/20)	A (20/20)	IV	A (2/2)	A (2/2)
C	V (5/5)	V (5/5)	II	V (1/1)	V (1/1)
–	–	–	III	V (1/1)	A (1/1)

*A* avirulent, *V* virulent

^a^Ratio in parenthesis indicates number of isolates with the corresponding phenotypes over the number of isolates within the given haplotype

A surprising feature for *Avr1k* was the presence of a frameshift mutation leading to an early stop codon in both haplotypes B and C, similar to the one reported by Song et al. [3]. If the truncation of the *Avr1k* protein makes it unrecognizable by *Rps1k*, this mutation should lead to a phenotype of virulence although isolates with haplotype B were avirulent. This phenomenon can be explained by the fact that the latter isolates share the same haplotype for *Avr1b*, which is seemingly recognized by *Rps1k.* Concerning the *Avr1b*/*Avr1k* interaction, it would be interesting to further study isolates that only show virulence to *Rps1b* or *Rps1k* to see if this pattern has evolved new or unusual haplotypes.

For three of the 31 isolates tested, deletion of *Avr1c* led to an expected virulence to plants carrying *Rps1c*. However, as with *Avr1b*, our data for *Avr1c* gave contrasting results of virulence when phenotyping the isolates with the haplotype of the reference genome (haplotype A). Re-phenotyping of the reference isolate confirmed a reaction of virulence in association with haplotype A. This suggests that *Avr1c*, as previously described, does not lead to a reaction of incompatibility with *Rps1c*, a situation that may explain why the efficacy of *Rps1c* has been described as unstable in the field [32]. Incidentally, Na et al. [10], who first identified *Avr1c*, also observed some discrepancy when phenotyping *P. sojae* isolates containing *Avr1c*, a situation they attributed mostly to gene silencing. On the basis of that suggestion, we further analyzed those isolates. Of the three remaining outliers following the phenotyping with the hydroponic assay, all isolates were associated with *Avr1c* and were virulent against soybean lines carrying *Rps1c* while associated with a haplotype that should confer an avirulent reaction. Expression analysis showed that *Avr1c* was significantly less expressed in these outliers compared to avirulent isolates presenting the same haplotype, which would explain the phenotypes observed. From a functional point of view, we hypothesized that this lower expression could find its origin in genomic variations. Incidentally, genome-wide sequence comparison revealed the deletion of a gene from the Sin3 family for one of the outliers, and deletion of the putative avirulence gene *Avh220* for another. These results offer a potential explanation for the transient expression of the avirulence gene and propose the implication of new genes in the virulence of *P. sojae* to *Rps1c*. These findings were only made possible because of the extensive whole genome sequencing analyses. Further investigations are needed to confirm that these two genes are interacting with *Rps1c*, but their nature offers a priori evidence of their implication in virulence. Indeed, the protein encoded by the deleted gene from the Sin3 family is recognized as a regulator of transcription [33]. Computational prediction for *Avh220*, the second gene found to be deleted in one isolate, suggests it is a putative RXLR effector with a potential role in virulence. The mechanism by which the sole remaining outlier, isolate 45B, succeeds in escaping *Rps1c* is still unclear. The many unique mutations found for this isolate do not seem to be linked to any factors related to virulence, but the possibility that this can lead to an epistatic interaction of one or many genes with the *Avr1c* gene cannot be completely dismissed. Epigenetic mechanisms might also be involved in the gain of virulence on *Rps1c* plants for this isolate. Another interesting aspect of *Avr1c* was the discovery a new allele (haplotype D) that shared many similarities with *Avr1a* sequences [10]. It is well known that *Avr1a* and *Avr1c* are closely related but reads from this allele were distinct from those that aligned against *Avr1a*, which would rule out the possibility of misalignment. Considering that *Avr1a* and *Avr1c* are often subjected to deletion, one could speculate that they are in the presence of DNA repair although proof for this process is lacking in *P. sojae*. Finally, a rare case of heterozygous variants was observed with two isolates (haplotype C). Because this heterozygosity is not encountered all across the gene region for those isolates, we excluded the presence of two different alleles as a result of sexual segregation but attributed it instead to the observed duplication of the *Avr1c* gene for those two isolates, resulting in presence of reads from both copies of *Avr1c* on the same locus following alignment on the reference genome.

A complete deletion of the *Avr1d* gene was also observed in some isolates but, unlike the case of *Avr1a*, a constant phenotype of virulence was associated with this deletion. An absence of coverage along a 2.2-kb segment with another upstream deletion of 0.8 kb, separated by a segment of 177 bp, including the *Avr1d* gene, was indeed revealed through our data. Previously, a deletion/virulence link for *Avr1d* was also reported by Na et al. [34] with the distinction that the latter group observed an absence of read coverage along a shorter segment of 1.5 kb in the isolates studied. Over time, it will be interesting to determine if the difference can be explained by an evolving zone of deletion or simply a different variant.

The haplotype analysis for *Avr3a* has revealed two distinct alleles, and a distinctive phenotyping response separating these two haplotypes, with no outlier. In addition to the discriminant haplotypes, all virulent isolates contained only one copy of the gene while the avirulent isolates contained between two and four copies, in contrast to previous results that reported exclusively four copies in avirulent isolates [13]. The haplotypes were similar to the ones described by Dong et al. [11]. In contrast, two SNPs reported in the earlier study did not appear in any of the isolates tested, although they do not affect the haplotype sequences.

In the case of *Avr*6, two distinct haplotypes emerged clearly delineating the interactions of compatibility and incompatibility once the isolates were re-phenotyped. Because of our extensive coverage, we were able report a unique SNPs and a deletion of 15 bp further upstream that represents a clear discriminant zone between virulent and avirulent isolates. SNPs closest to the gene were also reported in *P. sojae* isolates by Dou et al. [12].

## Conclusions

In conclusion, we took advantage of a new phenotyping procedure and WGS of 31 *P. sojae* isolates representative of the genetic diversity found in Canadian fields to conduct an exhaustive association analysis of phenotype and genotype for a total of 217 interactions. Our results identified new variants and new properties of some Avr effectors and refined the phenotypes associated with each variant to show that genomic signatures provided a near perfect prediction of phenotypes. We further suggest that the virulence model previously described for *Avr1c* should be reassessed.

## Methods

### Plant material and *Phytophthora sojae* isolates

A total of 31 isolates of *P*. *sojae* were selected on the basis of their diverse pathotypes for seven avirulence genes (1a, 1b, 1c, 1d, 1k, 3a and 6) and their prevalence (80%) among the races found in a collection of 275 isolates sampled across Ontario (Canada) between 2010 and 2012 and retrieved from Xue et al. [29]. Whenever possible, three isolates of the same race were used for analysis (Table 2). The reference strain P6497 was obtained from Dr. Mark Gijzen (Agriculture and Agri-food Canada, London, Ontario). Each of the 31 isolates was previously characterized for the presence of *Avr* genes using the hypocotyl wound-inoculation technique [29] where a set of eight differential soybean lines were used, each containing a single resistance *Rps* gene (*Rps1a*, *Rps1b*, *Rps1c*, *Rps1d*, *Rps1k*, *Rps3a*, *Rps6*, and *Rps7*), and “Williams” (*rps*) as a universal susceptible check.

**Table 2:** Races and associated pathotypes of *Phytophthora sojae* isolates characterized in this study, as determined by hypocotyl wounding inoculation [29]

Race	Pathotype	Number of isolates	Isolates IDs
1	7	2	1A, 1C
3	1a,7	3	3A, 3B, 3C
4	1a, 1c, 7	3	4A, 4B, 4C
5	1a, 1c, 6, 7	3	5A, 5B, 5C
7	1a, 3a, 6, 7	3	7A, 7B, 7C
8	1a, 1d, 6, 7	3	8A, 8B, 8C
9	1a, 6, 7	3	9A, 9B, 9C
22	1a, 1c, 3a, 6, 7	1	22
25	1a, 1b, 1c, 1k, 7	3	25B, 25C
25 + 1d	1a, 1b, 1c, 1d, 1k, 7		25D
28	1a, 1b, 1k, 7	3	28A, 28B, 28C
43	1a, 1c, 1d, 7	1	43
45	1a, 1b, 1c, 1k, 6, 7	3	45A, 45B, 45C

### DNA extraction and sequencing

DNA was extracted for each of the 31 isolates using the E.Z.N.A. Plant DNA Kit (Omega Bio-Tek Inc., Norcross, GA, USA). The DNA quantity and quality was assessed using a NanoDrop ND-1000 spectrophotometer (NanoDrop technologies). Each sample was normalized to 10 ng/μL for sequencing library construction using the NEBNext Ultra II DNA Library Prep Kit for Illumina (New England BioLabs Inc., Ipswich, MA, USA). Library quality was determined using the Agilent 2100 Bioanalyzer (Agilent Technologies). An average fragment size of approximately 650 bp was observed among all 31 individual samples. Paired-end, 250-bp sequencing was performed on an Illumina HiSeq 2500 (CHU, Québec, Canada).

### Reads alignment to the reference genome

Quality of the reads obtained from sequencing was checked using FastQC (Babraham Institute, Cambridge, UK). Reads were processed using Trimmomatic [35] to remove adapter sequences and bases with a Phred score below 20 (using the Phred + 33 quality score). Trimmed reads were aligned against the *P. sojae* reference genome V3.0 [27] using the Burrows-Wheeler Transform Alignment (BWA) software package v0.7.13 [36].

### Phylogenetic analysis

Phylogenetic inference of the isolates was made based on variant data obtained from the whole genome resequencing and a subset of variants identified within the region of seven *Avr* genes (1a, 1b, 1c, 1d, 1k, 3a, 6). The phylogenetic tree was developed by using a neighbor-joining method in Tassel software [37] and then visualized using MEGA 6.0 software tool [38]. Bootstrapping (500 replicates) was used to calculate the percentage of replicate trees in which the associated taxa were clustered together.

### Haplotype analysis

Haplotype analysis was done using a systematic approach. For every *Avr* gene studied, we began by searching for evidence of structural variation, namely presence/absence polymorphisms and copy number variation. We then examined nucleotide variation (SNPs or indels) within the genic regions that could be expected to lead to a loss of activity or that defined a specific haplotype that could be associated with the virulence phenotype. When further analysis was needed to find discriminant haplotypes, we also surveyed mutations in the vicinity of the gene. Once derived haplotypes were established, if discrepancies occurred between the observed genotype and phenotype for some isolates, virulence testing was performed using a hydroponic assay (see below). If the phenotype was still incongruent with the genotype for these isolates, as with *Avr1c*, we measured *Avr* gene expression to see if changes in transcript abundance could explain a gain of virulence. In parallel, targeted re-sequencing of the gene under investigation was done to check for mutation or contamination in the isolates that could have occurred in the time elapsed between DNA isolation for WGS and the ensuing virulence test. Targeted re-sequencing was also carried out for *Avr1b* and *Avr1k* in isolates and in the reference strain P6497 that had a discordance between the genotype and the phenotype.

### Presence/absence polymorphisms and copy number variation

To detect loss of avirulence genes in some isolates from the reference genome (presence/absence polymorphisms), we calculated the breadth of coverage for each gene, corresponding to the percentage of nucleotides with at least one mapped read (1× coverage), as per Raffaele et al. [39]. If the value of the breadth of coverage was below 80%, the gene was considered to be absent. For detection of copy number variation (CNV), we compared the average depth of coverage for each locus in every isolate and normalized the counts using the mean coverage of the genic region in every isolate.

### Variant detection

Variant calling was done using the Genome Analysis Toolkit (GATK) [40], a variant calling pipeline based on GATK’s best practices. The resulting raw vcf file was quality filtered using the vcfR package [41]. For haplotype visualization, a simple visual inspection was sufficient in most cases, but a custom script developed at Université Laval was used in other cases, based on a gene-centric haplotyping process that aims to select only markers in the vicinity of a gene that are found to be in strong linkage disequilibrium (LD).

### Virulence screening using the hydroponic assay

Whenever an isolate or P6497 had a phenotype predicted by the hypocotyl assay [29] discordant from the other isolates within a given haplotype, this isolate was re-phenotyped using a hydroponic assay, in which zoospores are inoculated directly into the hydroponic nutrient solution [26]. For this purpose, the isolate was tested against the appropriate differential line with three to six plants depending on the number of outliers to be tested within a given haplotype and the hydroponic system capacity for every replicate together with a susceptible control cultivar not carrying the appropriate *Rps* gene, a resistant control cultivar and a number of control isolates (see Additional file 3). Phenotypic responses for resistance or susceptibility were recorded at 14 days post-inoculation.

### Expression analysis

Total RNA was extracted from 7-day-old *P. sojae*-infected soybean roots using the Trizol reagent followed by purification using the Qiagen RNeasy Mini kit (Valencia, CA, USA). The RNA samples were treated with DNase I enzyme to remove any contaminating DNA. A total of 3 μg RNA from each sample were used to synthesize single-stranded cDNA using oligo-dT primed reverse transcription and Superscript II reverse transcriptase (Invitrogen™, Carlsbad, CA, USA) following the manufacturer’s protocol. Primers for the quantitative reverse transcription PCR (qPCR) analysis were designed using PrimerQuest tool and the intercalating dyes design option (Additional file 4; Coralville, IA, USA). Four biological replications were used for the expression analysis. Expression analysis was carried out for *Avr* genes in both avirulent and virulent isolates using the iQ™ SYBR® Green Supermix (Bio-Rad, Hercules, CA, USA) and a MIC qPCR thermocycler machine (Bio Molecular Systems, Upper Coomera, Queensland, Australia). The PCR profile consisted of an initial activation of 95 °C for 3 min, followed by 40 cycles of 95 °C for 15 s and 60 °C for 45 s. After cycling, dissociation curve analysis (with an initial hold of 95 °C for 10 s followed by a subsequent temperature increase from 55 to 95 °C at 0.5 °C/s) was performed to confirm the absence of nonspecific amplification. Actin was used as a constitutively expressed reference transcript. Relative quantification analysis was performed using the MIC-qPCR software which uses the LinRegPCR method developed by Ruijter et al. [42] and the Relative Expression Software Tool (REST) for statistical significance [43].

### Confirmation of haplotype variation using sanger sequencing

The isolates were freshly grown in V8 agar media for 7 days under controlled conditions followed by DNA extraction. Regions spanning the *Avr* genes were amplified using specific sets of primers (Additional file 3). The PCR profile was initial denaturation at 98°C for 30 s followed by 35 cycles of denaturation at 98°C for 10 s, annealing at 60°C for 30 s and extension at 72°C for 2 min, and the final extension at 72°C for 10 min. The PCR products were purified using the QIAquick PCR purification kit (Qiagen, Valencia, CA, USA) followed by sequencing on an Applied Biosystems sequencer (ABI 3730xl DNA Analyze) located at the CHU, Quebec, Canada. The sequencing results were analyzed using the SeqMan program implemented in the DNASTAR Lasergene software (Madison, WI, USA).

## Additional files

Additional file 1: All variants (SNPs and indels) found among 31 isolate of *Phytophthora sojae* for seven *Avr* genes. (XLSX 54 kb)

Additional file 2: Sequence alignment of Sin3 transcription factor showing deletion in 5C isolate. (XLSX 284 kb)

Additional file 3: Phenotypic responses of all isolates tested with the hydroponic assay. (PDF 40 kb)

Additional file 4: Primer sequences used for real time PCR and Sanger sequencing. (XLSX 16 kb)

Additional file 5: Individual values for relative expression of the *Phytophthora sojae Avr1c* gene measured by real-time quantitative PCR in avirulent and virulent isolates. (PDF 48 kb)

## Abbreviations

*Avr*: Avirulence
bp: Base pairs
*CNV*: *Copy number variation*
DLs: Differential lines
LD: Linkage disequilibrium
NILs: Near-isogenic lines
NLR: Nucleotide-binding domain and leucine-rich repeat region
ORF: Open-reading frame
*Rps*: Resistance to *P. sojae* genes
SNPs: Single nucleotide polymorphisms
WGS: Whole genome sequencing
