# Supplementary material for: Stable predictive markers for Phytophthora sojae avirulence genes that impair infection of soybean uncovered by whole genome sequencing of 31 isolates
Source: BMC Biol. 2018 Jul 26;16:80. doi: 10.1186/s12915-018-0549-9 (PMC6060493; doi:10.1186/s12915-018-0549-9)

**Additional File 2** : Sequence alignment of Sin3 transcription factor showing deletion in 5C isolate.

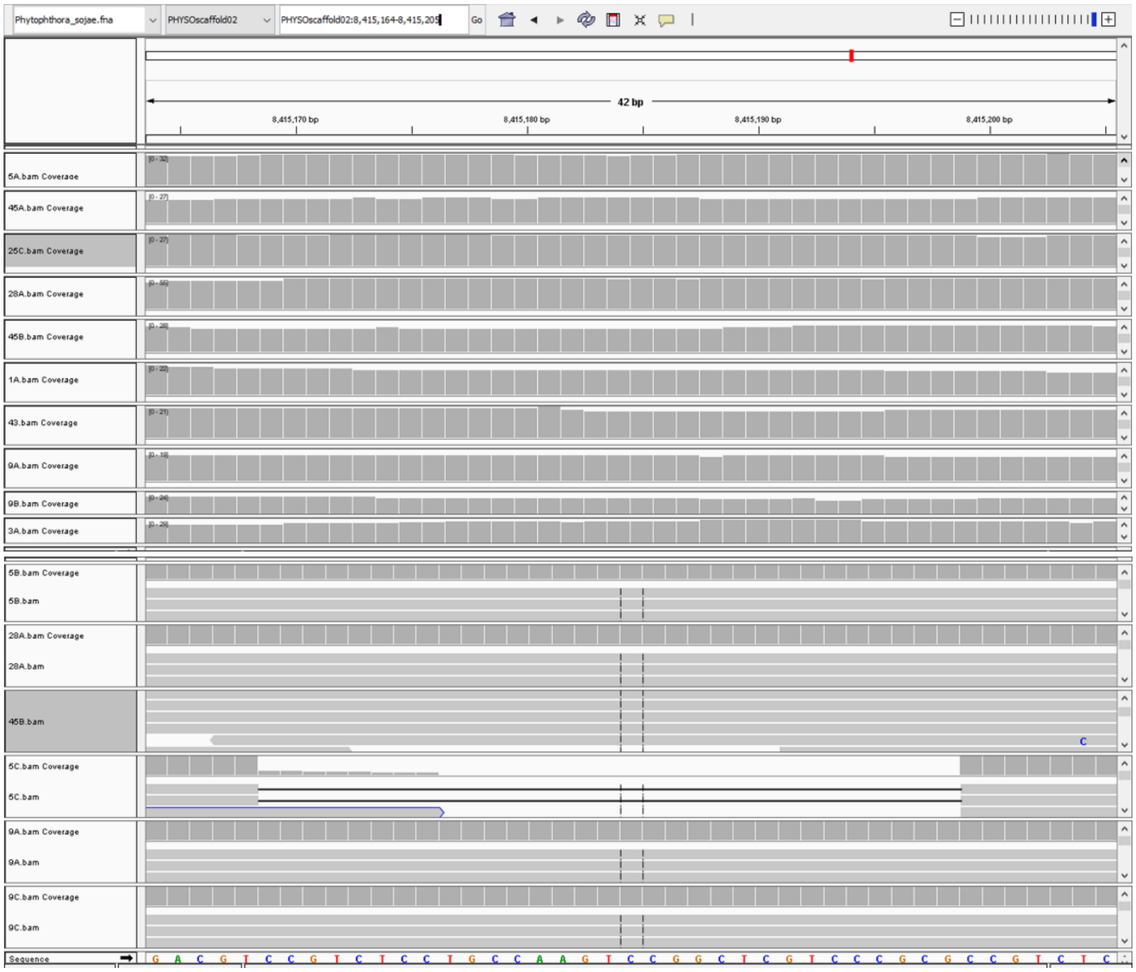

Supplement: Supplementary file 2 — Sequence alignment of Sin3 transcription factor showing deletion in 5C isolate. (XLSX 284 kb) [file 12915_2018_549_MOESM2_ESM.pdf]
