# Supplementary material for: Stable predictive markers for Phytophthora sojae avirulence genes that impair infection of soybean uncovered by whole genome sequencing of 31 isolates
Source: BMC Biol. 2018 Jul 26;16:80. doi: 10.1186/s12915-018-0549-9 (PMC6060493; doi:10.1186/s12915-018-0549-9)
Supplement: Supplementary file 4 — Primer sequences used for real time PCR and Sanger sequencing. (XLSX 16 kb) [file 12915_2018_549_MOESM4_ESM.pdf]

**Additional File 4** : Primer sequences used for real time PCR and sanger sequencing

| Gene | Forward sequence | Reverse sequence | Amplicon size |
|---|---|---|---|
| Avr1c (qPCR) | TGTATTCCACTTGAGAGGTCATTAG | CTGGTACAGACGATTCGAGAAC | 103 |
| Avr1b (qPCR) | TACGAGAAGTGGGCAAAGAAG | GGTGAAAGGTGTATCCGTTGTA | 118 |
| Avr1k (qPCR) | CCCGACTTGTCACTACTTCTC | CGTCATGCCACATACCAAAC | 100 |
| Actin (qPCR) | GCCACCACCTTGATCTTCAT | AAGGACCTGTACTGCAACATC | 122 |
| Tubulin (qPCR) | CGGTATGGGTACGCTTCTTATC | GACAGCGTAGCGTTGTAGG | 129 |
| Avr1c | CCGCATCATCATCTTCTTCTG | AGGAACCGACGAGCCTTGAG | 2012 |
| Avr1c-seq | TGATTGGGTCACACAAATATCC | | |
| Avr1b | CCTTCTGAGTCATGAGTTGCTG | GAGCAACGACAGGTTTTTAAGC | 5001 |
| Avr1k | TGTAACTTCACCGCCGAATATC | CGAGGTTTTAGGTCGTCTTTTC | 5001 |
| Avr1b-seq1F (nested) | TCATACAGAAGAGCATGTGCG | | |
| Avr1b-seq2F (nested) | TGAACAGCAAAAAGATCTGAACA | | |
| Avr1b-seq3F (nested) | AGGTGGCGCAAGTGAGAG | | |
| Avr1b-seq4F (nested) | TGTGGCTACATGTGAGAGCAG | | |
| Avr1b-seq5F (nested) | AAAAACAATAATACAATCCAGAGCA | | |
| Avr1b-seq6F (nested) | CAGACCCGAGTTTAGCGAAT | | |
| Avr1b-seq7F (nested) | CTATTTGCCTACCAGCCAATC | | |
